# Top-down modulation of ventral occipito-temporal responses during visual word recognition

**DOI:** 10.1016/j.neuroimage.2011.01.001

**Published:** 2011-04-01

**Authors:** Tae Twomey, Keith J. Kawabata Duncan, Cathy J. Price, Joseph T. Devlin

**Affiliations:** a Cognitive, Perceptual & Brain Sciences, University College London, Gower Street, London, WC1E 6BT, UK; b Wellcome Trust Centre for Neuroimaging, 12 Queen Square, London WC1N 3BG, UK

**Keywords:** Reading, Fusiform gyrus, fMRI, Lexical decision, Feedback

## Abstract

Although interactivity is considered a fundamental principle of cognitive (and computational) models of reading, it has received far less attention in neural models of reading that instead focus on serial stages of feed-forward processing from visual input to orthographic processing to accessing the corresponding phonological and semantic information. In particular, the left ventral occipito-temporal (vOT) cortex is proposed to be the first stage where visual word recognition occurs prior to accessing nonvisual information such as semantics and phonology. We used functional magnetic resonance imaging (fMRI) to investigate whether there is evidence that activation in vOT is influenced top-down by the interaction of visual and nonvisual properties of the stimuli during visual word recognition tasks. Participants performed two different types of lexical decision tasks that focused on either visual or nonvisual properties of the word or word-like stimuli. The design allowed us to investigate how vOT activation during visual word recognition was influenced by a task change to the same stimuli and by a stimulus change during the same task. We found both stimulus- and task-driven modulation of vOT activation that can only be explained by top-down processing of nonvisual aspects of the task and stimuli. Our results are consistent with the hypothesis that vOT acts as an interface linking visual form with nonvisual processing in both bottom up and top down directions. Such interactive processing at the neural level is in agreement with cognitive and computational models of reading but challenges some of the assumptions made by current neuro-anatomical models of reading.

## Research Highlights

►Activation in left vOT was modulated by task, even when stimuli were held constant. ►Activation in vOT was also modulated by stimulus differences. ►Activation in vOT could not be predicted by differences in response times. ►Top-down input to vOT is required to explain these effects.

## Introduction

Although cognitive models of reading emphasize the importance of interactive processing during visual word recognition, most neuro-anatomical models of reading have focused on the feed-forward flow of information. In the classic neurological model of reading, for example, visual input arrives at the occipital pole and projects to the angular gyrus where visual word forms are stored (Dejerine, 1891, 1892). These then link to auditory word forms in the posterior superior temporal lobe (i.e. Wernicke's area) and from there to articulatory motor patterns in the inferior frontal gyrus (i.e. Broca's area). In this linear fashion, a written word is recognized, converted into a sound then motor pattern, and read aloud. More recent studies elaborate additional anatomical territories (Bitan et al., 2009; Dehaene et al., 2005; Frost et al., 2008; Price and Mechelli, 2005), allow for multiple parallel pathways (Devlin, 2008; Mechelli et al., 2005), and characterize the functional contributions of the component regions differently (Shaywitz and Shaywitz, 2008). Even so, most neural models of reading continue to involve an essentially feed-forward, staged processing dynamic (Dehaene et al., 2005; Kronbichler et al., 2004).

At a behavioural level it is well established that reading requires interaction between visual and nonvisual properties of the written stimulus. A classic example is the “word superiority effect” where there is a perceptual advantage for identifying letters in words relative to visually matched letter strings that do not form words (McClelland and Rumelhart, 1981). The fact that letter detection is affected by whether or not the stimulus is a word – namely, by information not present in the visual display – illustrates that this information is automatically retrieved and fed back to affect visual processing. Although a purely feed-forward account of the word superiority effect has been proposed (Norris et al., 2000), this effect is only one source of evidence for interactivity during visual word processing. Another clear example is the finding that when participants make lexical decisions (i.e. decide whether a letter string forms a real word), they are slower to reject an item that sounds like a word (e.g. “brane”) than one that that does not (e.g. “brate”, McCann et al., 1988). This effect illustrates that automatic retrieval of phonological and/or semantic information that is not essential for task performance can nonetheless affect behaviour. These, and other similar observations (Frost, 1998; Reimer et al., 2008; Rosson, 1983; Smith and Besner, 2001), demonstrate the need for feedback connections linking nonvisual to visual information processing, thus creating an interactive (rather than feed-forward) system for visual word recognition (Coltheart et al., 2001; Harm and Seidenberg, 2004; Jacobs et al., 2003; McClelland and Rumelhart, 1981; Perry et al., 2007; Plaut et al., 1996; Rumelhart and McClelland, 1982).

This discrepancy between cognitive interactivity, on the one hand, and serial, feed-forward neuro-anatomical models, on the other, is particularly relevant to theories of ventral occipito-temporal (vOT) cortex functioning during reading. This region of extrastriate visual cortex is consistently engaged during visual word recognition and damage to the area can result in severe reading deficits (Behrmann et al., 1998; Cohen et al., 2000; Leff et al., 2001; Philipose et al., 2007; Starrfelt et al., 2009). As a result, vOT is thought to play an important role in orthographic processing (McCandliss et al., 2003; Price and Mechelli, 2005). One influential account suggests that visual information is encoded through a sequence of stages, from simple feature detectors located in early visual cortex, to letter detectors in V4, to bigram detectors in vOT, and then on to whole word detectors located even more anteriorally in the temporal lobe (Dehaene et al., 2005). In other words, orthographic information is progressively extracted following hierarchical, feed-forward steps that detect progressively more complex visual features. Although vOT receives primarily bottom-up visual information, the authors note that certain attentional manipulations can also provide a top-down signal such as when participants are asked to visualize written words (Cohen et al., 2004, 2002). For example, although auditory words do not typically engage vOT (Dehaene et al., 2002; Spitsyna et al., 2006), a recent study found that when participants selectively attended to auditory words it produced activation within the region (Yoncheva et al., 2010). This type of top-down attentional control, however, is fundamentally different from the automatic interactions between visual and non-visual (e.g. phonological or semantic) properties of a visual stimulus such as a word. These interactions are the type of top-down processing, carried in the feedback connections, that are crucial to cognitive and computational models of reading (Coltheart et al., 2001; Harm and Seidenberg, 2004; Jacobs et al., 2003; Perry et al., 2007; Plaut et al., 1996) but missing from most neuro-anatomic models (e.g. Cohen et al., 2002; Dehaene et al., 2005; Kronbichler et al., 2004). An alternative neural model suggests that vOT continuously and automatically interacts with other regions during reading, acting as an interface associating bottom-up visual form information critical for orthographic processing with top-down higher order linguistic properties of the stimuli (Cai et al., 2010; Devlin et al., 2006; Hillis et al., 2005; Kherif et al., 2011; Nakamura et al., 2002; Price and Friston, 2005; Xue et al., 2006).

Ideally, evidence for the direction of information flow in the reading network requires effective connectivity analyses that measure how activity in one region is influenced by activity in other regions. Such inferences are possible with dynamic causal modelling (DCM) of fMRI data (Friston et al., 2003), however, current implementations of this technique can only test the interactions among a limited number of regions. DCM therefore relies on knowing, *a priori*, where top down inputs to vOT are coming from. Several previous studies have used DCM to investigate functional connectivity between vOT and other parts of the reading system (Bitan et al., 2005, 2006, 2009; Booth et al., 2008; Cao et al., 2008; Heim et al., 2009; Mechelli et al., 2005; Nakamura et al., 2007; Seghier and Price, 2010). In all cases, however, the reports emphasize the feed-forward processing from vOT. For example, Booth et al. (2008) report that even though there was weak evidence for increased top down modulations from left Heschl's gyrus to the left fusiform during their auditory spelling task, this was not detected during the visual spelling task.

Despite the emphasis on feed-forward processing from vOT, other fMRI studies have reported data that is best interpreted in terms of interactions between language processing and visual word form processing in vOT. For example, Kherif et al. (2011) reported that vOT activation for reading object names was suppressed when primed with a masked picture of the same object relative to a masked picture of a different object, suggesting that non-visual processing that is common to words and pictures (e.g. semantics and phonology) was influencing vOT activation. Crucially, these could not be expectation-driven attentional effects because the visual masked priming paradigm precluded subjects from conscious awareness of the primes. Instead, these priming effects provide strong evidence of automatic interactions between the different types of visual and nonvisual information important for reading words.

The aim of this study was to investigate whether activation in vOT during visual word recognition is influenced by top-down nonvisual information. Participants performed two different types of lexical decision tasks which focused on either visual (i.e. orthographic) or nonvisual (i.e. phonological or semantic) properties of the stimulus. In one, participants were asked to decide whether the letter string was a real English word or not. Half of the stimuli were words (e.g. “brain”) and the other half were pseudohomophones — that is, pronounceable nonwords that sound like real words such as “brane.” When performing this task, participants had to focus on the visual properties of the stimuli to make the correct response since phonological and semantic properties of the stimuli would not differentiate a real word from a pseudohomophone. In the other task, participants were asked to decide whether the letter string on the screen sounded like a real word or not. Half of the stimuli were pseudohomophones (e.g. “beest”) and the other half were pseudowords (e.g. “beal”). In this task, participants had to focus on the phonological (and possibly semantic) properties of the stimuli to make the correct response since the visual properties of the stimuli were insufficient to perform the task as neither type of stimuli was visually a word.

Unlike previous studies that only used a single task (“Does the item sound like a word?” Bruno et al., 2008; Kronbichler et al., 2007; van der Mark et al., 2009), our design enabled us to examine two different types of top-down processing, namely stimulus-driven and task-driven effects. Stimulus effects were evaluated within task by carefully matching the stimuli on a range of visual properties (see below) such that if processing is primarily feed-forward, vOT activation would be expected to be comparable across conditions. If, on the other hand, the region also receives feedback from higher order areas, then nonvisual properties would be expected to significantly modulate vOT activation levels. Task effects were evaluated by holding the stimulus constant and comparing the activations to pseudohomophones across tasks. Feed-forward accounts predict that pseudohomophone activations in vOT would either be comparable across tasks (as the stimuli were carefully matched) or possibly increased for orthographic relative to phonological lexical decisions. In the case of a purely feed-forward account, increased activation in vOT during the orthographic relative to phonological task could be based solely on increased local processing demands without requiring any feedback interactions. In contrast, increased activation in vOT during the phonological relative to orthographic task would indicate greater interactions between regions involved in phonological and orthographic processing, consistent with feedback connections linking these areas. Here we tested these predictions using functional magnetic resonance imaging.

## Material and methods

### Participants

20 monolingual native English speakers (11M, 9F) participated in this study. All were from the British Home Counties (i.e. southern England) with the same regional accent, which was important for consistent pronunciation of nonwords. The data from four participants were excluded in total. One subject was excluded due to excessive motion inside the scanner (> 3 mm); one subject was excluded due to task performance that was not significantly above chance (i.e. < 65% accuracy); and two subjects were excluded because unexpected structural abnormalities were present in their T1 images. The ages of the remaining 16 (9M, 7F) participants ranged from 19 to 43 (M = 30). All were right-handed and none reported any history of neurological problems or reading difficulties. The experiment was approved by the NHS Berkshire Research Ethics Committee.

### Tasks and stimuli

**Fig. 1:**
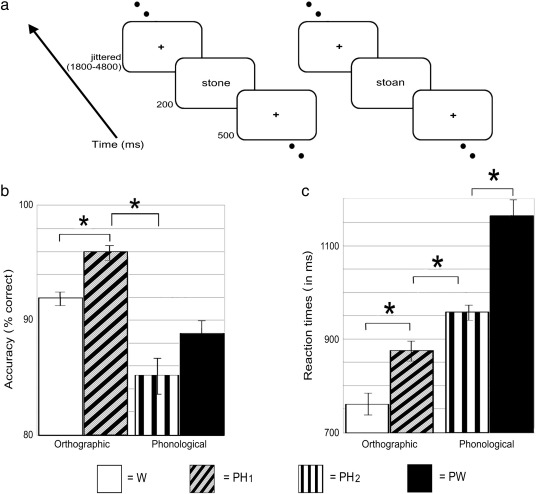
a) Schematized task. Each trial began with a fixation cross presented for 500 ms. A stimulus was then presented for 200 ms, followed by a jittered inter-stimulus interval of 1800–4800 ms (M = 3300 ms). b) Mean accuracy and c) reaction times for all four conditions. An * indicates *p* < .05. Abbrev: W = words (orthographic task), PH_1_ = pseudohomophones (orthographic task), PH_2_ = pseudohomophones (phonological task) and PW = pseudowords (phonological task).

There were two lexical decision tasks that forced participants to attend to different aspects of the stimuli. The first task emphasized visual over nonvisual properties of the stimuli whereas the second emphasized nonvisual over visual information. Consequently, we will refer to these as the ‘orthographic’ and ‘phonological’ lexical decision tasks, respectively. For both tasks, participants viewed a string of letters presented sequentially. For the orthographic lexical decision task, participants were instructed to decide whether the string formed an existing English word or not. For the phonological lexical decision task, participants were asked to decide whether the string sounded like an existing English word or not (Fig. 1a).

A behavioural pre-test was conducted with an independent set of 52 (28M, 24F) participants to pilot the stimuli and establish baseline performance in a reasonably large sample. All participants were monolingual native English speakers aged 17 to 69 (M = 27). For the orthographic lexical decision task, there was no significant difference in accuracy between words and pseudohomophones (93.7% vs. 93.1%, *t*(51) = .50, *p* = .622) but responses to words were significantly faster (779 vs. 1052 ms, *t*(51) = 11.37, *p* < .001). For the phonological lexical decision task, responses to pseudohomophones were less accurate than to pseudowords (85.1% vs. 88.9%, *t*(51) = 2.02, *p* = .049) but were significantly faster (1061 vs. 1478, *t*(51) = 10.78, *p* < .001), possibly indicating a speed–accuracy tradeoff. Anecdotally it became clear that because the participants in this behavioural pilot study came from geographically diverse areas of the UK, different regional accents contributed additional variability to the phonological lexical decision task due to different pronunciations of nonwords. Even so, a fairly large sample size ensured an adequate estimate of baseline performance. Given the smaller sample used in the fMRI study, we chose to recruit from a more uniform population of accents to minimize this variability.

Following the behavioural pre-test, stimuli were revised to exclude ambiguous items and the final stimulus set used for the fMRI tasks was comprised of 48 stimuli in each condition (192 stimuli in total). Stimuli were all monosyllabic and balanced for the number of letters (M = 4.5, *F*(3,188) = 1.07, *p* = .364), frequency of single letters (M = 281379, *F*(3,188) = .196, *p* = .899), bigram frequency (M = 1553, *F*(3,188) = 1.52, *p* = .211), trigram frequency (M = 258, *F*(3,188) = 1.85, *p* = .141) and orthographic neighborhood (M = 6.1, *F*(3,188) = .13, *p* = .943) based on N-Watch (Davis, 2005). For the word condition, the mean frequency per million words of British English was 76 as derived from the Celex database (Baayen and Pipenbrook, 1995) and the mean familiarity rating was 430 and was calculated from the MRC Psycholinguistic Database (Coltheart, 1981). For each task, the full set of 96 stimuli was divided evenly into two runs of 48 trials. For the orthographic lexical decision task, we ensured that no pairs of a real word and its pseudohomophone (e.g. “brain” and “brane”) occurred in the same run in order to avoid any priming effects. A different set of pseudohomophones was used in the phonological lexical decision task to ensure that no stimulus was repeated across tasks in order to avoid any priming effects and to avoid switching response type from “no” (in orthographic task) to “yes” (in phonological task) for the identical stimuli. We will refer to these two sets of pseudohomophones as PH_1_ (orthographic task) and PH_2_ (phonological task) to emphasize the fact that the stimulus sets were independent. The base words of PH_1_ and PH_2_ were balanced for frequency (M = 59, *t*(58) = 1.10, *p* = .275) and familiarity (M = 457, *t*(85) = 1.40, *p* = .165) to ensure that if differences are observed between pseudohomophones across tasks, these are the result of task-differences rather than potential psycholinguistic confounds. The order of both tasks and stimulus sets within a task were fully counter-balanced across participants.

A mixed block and event-related design was used. Participants performed a 33 s block of trials which included both “yes” and “no” responses in a pseudorandomized order. These were separated by 15 s blocks of fixation which served as an implicit baseline. Each trial began with a fixation cross presented for 500 ms. A stimulus was then presented for 200 ms, followed by a jittered inter-stimulus interval of 1800–4800 ms (M = 3300 ms). Therefore, the average trial length was 4 s. Stimuli were presented in a block of 8 trials. Over a run, there were six blocks of task performance and five blocks of rest. Therefore, each run lasted 4.85 min and there were a total of four runs (two per task). Responses were made with a button press, using either the index or middle finger of their right hand to indicate “yes” and “no”. The response fingers were fully counter-balanced across participants. The stimuli were projected onto a screen and viewed via mirrors attached to the head coil. Participants practiced each task inside the scanner before the main runs began. No items that were used in the practice runs occurred during the main experiment.

### MRI acquisition

Whole-brain imaging was performed on a Siemens Avanto 1.5 T MR scanner at the Birkbeck-UCL Neuroimaging (BUCNI) Centre in London. The functional data were acquired with a gradient-echo EPI sequence (TR = 3000 ms; TE = 50 ms; FOV = 192 × 192; matrix = 64 × 64) giving a notional resolution of 3 × 3 × 3 mm. Each run consisted of 97 volumes and as a result, the four runs together took 19.4 min. In addition, a high-resolution anatomical scan was acquired (T1-weighted FLASH, TR = 12 ms; TE = 5.6 ms; 1 mm^3^ resolution).

### Analyses

Items whose accuracy was below 65% were excluded from all analyses (n = 10). RTs were recorded from the onset of the stimulus. To minimize the effect of outliers, median RTs for correct responses per condition per subject were used in the statistical analyses and no items were trimmed (Ulrich and Miller, 1994). Because the two tasks used different types of stimuli (words and pseudohomophones vs. pseudohomophones and pseudowords), the experimental design was not factorial. Consequently, the data were analysed using a repeated measures 1 × 4 analysis of variance (ANOVA) with Condition as the independent variable. For the behavioural data, accuracy and reaction times (RTs) were the dependent measures. Where Mauchly's test indicated significant non-sphericity in the data, a Greenhouse–Geisser correction was applied. When there was a main effect of Condition, planned comparisons used paired t-tests to evaluate differences between the two conditions per task to evaluate stimulus effects and between the two pseudohomophone conditions to evaluate task effects.

The imaging data were processed using FSL 4.0 (www.fmrib.ox.ac.uk/fsl). The first two volumes were discarded in order to allow for T1 equilibrium. The data were then realigned to remove small head movements (Jenkinson et al., 2002), smoothed with a 6 mm full width at half maximum Gaussian kernel, and pre-whitened to remove temporal autocorrelation (Woolrich et al., 2001). The pre-processed data from each subject were then entered into a first level statistical analysis and modelled as events using a general linear model. The two main regressors corresponded to the correct trials from the two task conditions (per task) and these were convolved with a double gamma canonical hemodynamic response function (Glover, 1999). Eight additional regressors-of-no-interest were added: i) errors trials (Murphy and Garavan, 2004), ii) six estimated motion parameters, and iii) reaction times (RTs). It is important to note that the inclusion of RTs in the model only accounts for first-order (i.e. linear) effects and therefore higher-order (i.e. polynomial) relations between effort (as indexed by RTs) and BOLD signal may remain. Nonetheless, simple correlations between effort and BOLD signal were treated as a covariate-of-no-interest in order to model systematic differences in effort between conditions seen in the behavioural pilot. To remove low frequency confounds, the data were high-pass filtered with a cut-off point of 100 s. The contrasts of interest at the first level were the two experimental conditions relative to fixation per task. First level results were registered to the Montreal Neurological Institute (MNI)-152 template using a 12 degree of freedom affine transformation (Jenkinson and Smith, 2001) and all subsequent analyses were conducted in the MNI standard space. A second level fixed-effects model combined the two first level runs into a single, subject-specific analysis (per task) which was then entered into a third level, mixed effects analysis to draw inferences at the population level (Beckmann et al., 2003; Woolrich et al., 2004).

The first analysis identified areas of activation that were common to all four conditions using a linear contrast to compute their mean activity (i.e. [1 1 1 1]) and inclusively masking it with each condition relative to fixation at *Z* > 3.1 (i.e., masking with [1 0 0 0], [0 1 0 0], [0 0 1 0], and [0 0 0 1]). A second analysis used a 1 × 4 ANOVA to identify areas showing significant differences across conditions (i.e. a main effect of Condition identified using an F-contrast). These were characterized by plotting the mean effect sizes per condition in a sphere (5 mm radius) centred on the peak coordinate.

Since the primary aim of this study was to investigate the top-down modulation on left vOT, we defined an *a priori* anatomical mask for this region. The main anatomical areas of interest are the occipito-temporal sulcus and adjacent regions on the crests of the fusiform and inferior temporal gyri: areas consistently activated by visual word recognition tasks (Bitan et al., 2007; Cai et al., 2010; Cohen et al., 2000; Devlin et al., 2006; Duncan et al., 2009; Fiez and Petersen, 1998; Frost et al., 2005; Herbster et al., 1997; Kronbichler et al., 2007; Price et al., 1996; Rumsey et al., 1997; Shaywitz et al., 2004; van der Mark et al., 2009). Because the precise coordinates vary along a rostro-caudal axis, standard space coordinates ranging from X = − 30 to − 54 and Y = − 45 to − 70 were used to delineate this region. In addition, the depth of the sulcus coupled with the fact the temporal lobe is angled downwards required a range of Z-coordinates as well (Z = − 30 to − 4). Together these coordinates describe a rectangular prism that conservatively encompass the anatomical regions-of-interest but also include parts of the cerebellum that were not of interest. Consequently these were manually removed from the mask. A small volume correction determined that a voxel threshold of *Z* > 3.2 corresponded to *p* < .05 after correcting for the number of independent comparisons within the region (Worsley et al., 1996) and this was used for all vOT analyses. With an unconstrained, whole brain search, a corrected voxel-wise *p*-value of .05 corresponded to *Z* > 4.6. To minimize Type II errors, we also report activations present at *Z* > 4.0 as trends.

## Results

### Behavioural results

The behavioural data (Fig. 1) demonstrated significant differences across Conditions for both accuracy (*F*(3,45) = 11.98, *p* < .001) and reaction times (*F*(1, 22) = 31.90, *p* < .001, with Greenhouse–Geisser correction). Moreover, Fig. 1 clearly shows evidence of both stimulus- and task-related differences. In the orthographic task, responses to words were less accurate (92% vs. 96%, *t*(15) = 2.98, *p* = .009) but faster (761 vs. 874 ms, *t*(15) = 6.76, *p* < .001) than responses to pseudohomophones. A similar pattern was present in the phonological task. Here, responses to pseudohomophones were numerically less accurate (85% vs. 89%, *t*(15) = 1.74, *p* = .102) but significantly faster (956 vs. 1162 ms, *t*(15) = 5.30, *p* < .001) than responses to pseudowords. In other words, like the behavioural pre-test, these results suggest that participants may have adopted a speed–accuracy trade-off within each task. Therefore, when analysing the imaging data, we considered only correct trials and explicitly modelled RTs on a trial-by-trial basis to account for these first order, systematic differences between conditions. In addition to these stimulus effects, there was also a significant task effect when comparing the pseudohomophone conditions. Responses were more accurate (96% vs. 85%, *t*(15) = 4.69, *p* < .001) and faster (874 vs. 956 ms, *t*(15) = 2.32, *p* = .035) when participants made orthographic relative to phonological lexical decisions. In summary, the behavioural results demonstrate both stimulus- and task-effects on behaviour, consistent with top-down influences in visual word recognition (McCann et al., 1988).

### Imaging results: Common system

**Fig. 2:**
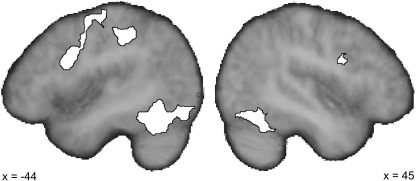
The brain areas commonly activated for all four conditions relative to fixation. Activations are thresholded at *Z* > 3.1 and shown as white areas (outlined in black) on two parasagittal slices through the mean structural image of the group in standard (i.e. MNI152) space.

We began by identifying the common system of regions activated by all four conditions (Fig. 2). As expected, there was strong bilateral activation in vOT centred on the posterior occipito-temporal sulcus that extended inferiorally into lobule VI of the cerebellum. In addition, there was bilateral activation in the early visual cortices of the calcarine sulcus, in the intraparietal sulcus, the deep frontal operculum and at the junction of the inferior frontal and precentral sulci. There was also left hemisphere activation in the pre-SMA, the anterior supramarginal gyrus and within sensori-motor cortices that included the omega-knob marker for the hand area (Yousry et al., 1997). In other words, these results correspond closely to previous lexical decision studies, validating the success of the task (Carreiras et al., 2007; Devlin et al., 2006; Fiebach et al., 2007; Gold et al., 2006; Kiehl et al., 1999; Mummery et al., 1999; Rumsey et al., 1997). Table 1 provides the full details of these activations and illustrates that for each region, there is activation in each of the four conditions. Presumably these reflect common aspects of the two tasks including not only visual word recognition, but also sustaining attention, maintaining a cognitive set and making manual responses.

The critical analysis, however, looked for activation differences across our four conditions reflecting the different top-down processing demands. Areas that were significantly affected by Condition were identified from the F-map of the one-way ANOVA and fell into two classes. The first set included ventral occipito-temporal cortex and pars opercularis (POp) — where activation was increased during all conditions relative to fixation. The second set included the angular gyrus, medial prefrontal cortex, and precuneus — areas showing significant deactivations. Although we report the second set of effects for completion, we focus on the top-down processing effects in our region of interest (vOT) and in POp which showed the same pattern of effects as vOT.

### Activations

**Fig. 3:**
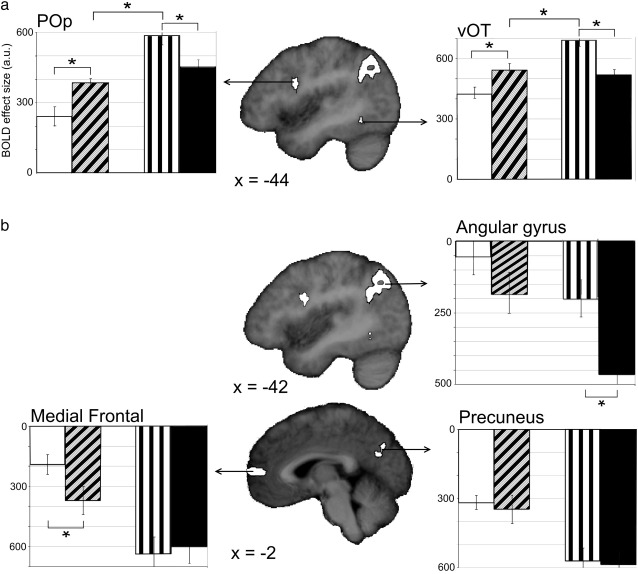
Regions whose activations differed across the four conditions. Also shown are bar plots of the BOLD signal per condition relative to fixation in each region. The conditions are illustrated using the same key as Fig. 1. a) The top panel illustrates stimulus- and task-dependent modulation of activation in left ventral occipito-temporal (vOT) cortex and left pars opercularis (POp). The BOLD response profile in these two regions was essentially identical and did not follow the RT profile (Fig. 1) and thus could not be explained solely in terms of effort. Note that the opercular activation was not part of the common activation seen at the junction of the inferior frontal and precentral sulci because words, unlike the other three conditions, were not significantly activated relative to fixation (*Z* = 1.6). b) The bottom panel illustrates significant differences across conditions due to deactivations and is consistent with stimulus- and task-independent responses seen in the default network. Statistical threshold = *p* < .05 (* = significant). Activations are thresholded at *Z* > 3.09 and only clusters with significant, or nearly significant, activations are shown (i.e. *Z* > 3.2 in the vOT region-of-interest or *Z* > 4.0 across the whole brain).

The most significant effect in the F-map was located in posterior occipito-temporal sulcus at [− 44, − 54, − 12; *Z* = 3.5], precisely in the region of the so-called “visual word form area” (Cohen et al., 2000, 2002; cf. Price and Devlin, 2003). Fig. 3a shows the region and illustrates how its BOLD signal response profile differed across the four conditions. Planned comparisons of vOT responses revealed that, within both tasks, there were significant stimulus effects. In the orthographic lexical decision task, there was greater activation for pseudohomophones than for words (*t*(15) = 2.23, *p* = .041) mirroring the RT pattern. In contrast, for phonological lexical decisions the effect sizes went in the opposite direction to the behavioural results, with significantly greater activation for pseudohomophones than pseudowords, (*t*(15) = 4.42, *p* < .001). Finally, the direct comparison of the two pseudohomophone conditions revealed significant task-related differences with greater activation in the phonological than the orthographic task (*t*(15) = 2.70, *p* = .017), once again mirroring the RT pattern.

This same pattern of activation was also observed in a region of left POp [− 51, + 10, + 16], although it was only a trend (*Z* = 4.3). As in vOT, there was a significantly greater activation for pseudohomophones relative to words in orthographic lexical decisions (*t*(15) = 2.92, *p* = .010), significantly more activation for pseudohomophones relative to pseudowords in phonological lexical decisions (*t*(15) = 3.01, *p* = .009) and a significantly more activation for pseudohomophones in the phonological task relative to those in the orthographic task (*t*(15) = 4.56, *p* < .001). In sum, both vOT and POp showed a similar pattern of activation, consistent with top-down modulation.

### Deactivations

A very different pattern of significant differences across conditions was observed within the left angular gyrus [− 42, − 65, + 47; *Z* = 4.9]. Here, all four conditions showed deactivation relative to fixation, and moreover, the magnitude of the deactivation corresponded to the amount of effort required, with the largest effects in conditions showing the longest RTs (Fig. 3b). The fact that the magnitude of the deactivations was greater in conditions with the longest RTs despite including RTs as a covariate-of-no-interest in the statistical model indicates a non-linear (e.g. higher order) relation between effort and BOLD signal reductions. Two additional areas showing a trend for significant differences across conditions also demonstrated deactivations relative to fixation, namely the medial prefrontal cortex [− 2, + 63, + 8; *Z* = 4.3] and the precuneus [− 4, − 65, + 29; *Z* = 4.1]. Together these three regions are often considered core components of the “default mode network” (Binder et al., 1999; Greicius et al., 2003; Mazoyer et al., 2001; Raichle et al., 2001; Raichle and Snyder, 2007; Shulman et al., 1997), which is consistent with the deactivations relative to fixation observed here. Indeed, greater deactivation within the default mode network has even been shown to correlate with increasing effort (Lin et al., in press).

## Discussion

The aim of this study was to investigate whether activation in vOT during commonly used word recognition tasks is influenced by top-down processing of nonvisual properties of the visual stimuli. We used words, pseudohomophones and pseudowords in two separate lexical decision tasks in order to manipulate the processing demands on visual and nonvisual aspects of the written stimuli. The findings demonstrated that activation in the left vOT (at x = − 44, y = − 54, z = − 12; the precise location of the so-called “visual word form area”) was significantly different across the four conditions and the pattern of activation here could not be predicted by differences in response times. In order to characterize the observed effect, we begin by discussing the stimulus effects within each task and then turn to the task effects seen for pseudohomophones.

Accurate performance on the orthographic task required participants to ignore nonvisual properties of the stimulus and focus instead on its specific visual form since all stimuli could be associated with phonological (and semantic) information. Here we found greater activation for pseudohomophones relative to words in vOT (Fig. 3), replicating previous studies (Bruno et al., 2008; Kronbichler et al., 2007; van der Mark et al., 2009). This finding is difficult to reconcile with a feed-forward account of progressively larger orthographic detectors (Dehaene et al., 2005) because words and pseudohomophones were carefully matched for pre-lexical visual properties such as letter, bigram and trigram frequencies. Kronbichler et al. (2004) suggested an alternative feed-forward hypothesis in which the visual forms of whole words are stored in vOT, presumably as word detectors analogous to the bigram detectors proposed by Dehaene et al. (2005). By this account, pseudohomophones partially activate multiple word detectors yielding greater activation than a single, fully-active word detector (Kronbichler et al., 2004). Although consistent with findings from our orthographic task, this explanation runs into difficulties explaining the results from the phonological task.

The phonological lexical decision task required that unfamiliar visual forms were ignored and instead focused on the phonological (and perhaps semantic) properties of the letter strings. Here we found a significantly greater activation for pseudohomophones relative to pseudowords. Moreover, this activation difference went in the opposite direction to the behavioural difference, effectively ruling out effort as a possible explanation and suggesting that the difference had to relate to processing the stimuli themselves. According to Kronbichler et al. (2004), both types of stimuli would be expected to partially activate word detectors to similar extents, yielding comparable activation levels for pseudowords and pseudohomophones. Clearly, this was not the case. Instead, pseudohomophones produced significantly greater activation than pseudowords in vOT despite being matched on their orthographic properties. As a result, this finding suggests that the difference in activation was most likely driven by nonvisual properties that differentiate the two conditions. Although both are pronounceable and therefore have an associated phonological pattern, these phonological patterns are only familiar for pseudohomophones where they correspond to existing words. Greater vOT activation may reflect the differential cost of integrating these nonvisual phonological and semantic properties with their visual forms via feed-back projections to vOT. In other words, the finding that nonvisual properties modulated activation in vOT demonstrates that this region does more than relay visual information forward to the language system; it interactively integrates bottom-up visual signals with top-down higher order information that is not present in the visual stimuli.

Given the theoretical importance of the finding, it is worth noting that two recent studies have also found greater vOT activation for pseudohomophones relative to pseudowords in a similar task (Bruno et al., 2008; van der Mark et al., 2009). Both studies used a similar phonological lexical decision task (“Does the item sound like a word?”), although their stimuli included real words (“taxi”) in addition to pseudohomophones (“taksi”) and pseudowords (“tazi”). In this design, real words benefit from a familiar orthographic pattern that facilitates “yes” responses relative to pseudohomophones and thus reduces vOT activation, consistent with the claim that lexical visual word forms are stored in the area (Kronbichler et al., 2007). Like the current study, van der Mark et al. (2009) reported significantly enhanced vOT activation for pseudohomophones relative to pseudowords which was also present numerically, but not reliably, in the study by Bruno et al. (2008). This effect, however, is difficult to reconcile within a lexical visual word form account (Kronbichler et al., 2004, 2007) without positing some form of feedback from non-visual properties of the stimuli that modulates vOT activation levels.

Finally, in addition to these stimuli-effects, we observed a significant effect of task on vOT activation when the stimuli were held constant, namely greater activation for pseudohomophones during phonological relative to orthographic lexical decisions. This novel finding is at odds with feed-forward accounts which predict either: i) no modulation in activations for pseudohomophones across tasks because the stimuli are the same in both cases or ii) greater activation for orthographic task due to increased orthographic processing. Because the stimuli were held constant (i.e. the two tasks used a carefully matched set of pseudohomophones), the change in vOT activation cannot be driven by the stimuli themselves but must instead be a consequence of the different nonvisual processing demands required by the two tasks. For instance, this task effect may reflect the additional phonological demands on decoding or assembly which is essential for the phonological task but not for the orthographic task (cf. Dietz et al., 2005). In other words, the increase seen during the phonological lexical decision task is an index of top-down modulation that is consistent with interactive accounts. The task effect can be explained by the interface account in terms of the greater demands on integrating bottom up visual processing with top down nonvisual information.

If correct, this hypothesis offers a single, principled explanation for the current findings and is consistent with previous studies whose results are difficult to explain without an interactive framework (Cai et al., 2010; Devlin et al., 2006; Kherif et al., 2011). In both the orthographic and phonological tasks, activation for pseudohomophones was greater than for words or pseudowords, respectively, indicating increased processing demands. Presumably, these increased demands are caused by the conflicting visual and nonvisual properties of pseudohomophones (Harm and Seidenberg, 2004). Pseudohomophones initially activate semantic information consistent with their phonological form, although this is rapidly suppressed (Harm and Seidenberg, 2004; Lukatela and Turvey, 1994). If vOT plays a role integrating this information, then the top-down semantic signal will conflict with the bottom-up visual information, requiring additional processing to suppress the inappropriate semantic pattern, thus increasing activation for pseudohomophones relative to words or pseudowords where there is no such conflict. In other words, it is precisely the integration of visual and nonvisual information that drives the activation observed in vOT. Furthermore, such conflict will have a greater effect on pseudohomophones during the phonological task relative to the orthographic task and this is precisely what we found. This interactivity between bottom-up visual information and top-down linguistic codes easily explains why vOT lateralization follows hemispheric language dominance in individuals (Cai et al., 2010) and can also account for nonvisual priming effects observed in vOT (Devlin et al., 2006; Kherif et al., 2011).

Could the current findings be explained by a different type of feed-forward account such as that of Norris et al. (2000)? According to this hypothesis, apparent top-down effects such as word superiority or pseudohomophone effects occur not at the level of processing the stimulus, but rather during the decision making process. Both functional neuroimaging and lesion-deficit studies with neurological patients have consistently associated decision making processes with prefrontal regions (Fleming et al., 2010; Walton et al., 2004; Weller et al., 2007), consistent with the stimulus- and task-driven modulation we observed in POp. This explanation runs into difficulty, however, accounting for the similar pattern of activation observed in vOT, a unimodal sensory area, unless of course it is due to feedback projections from prefrontal regions. In other words, the fact that effects we observed were present in the early perceptual stages of processing is incompatible with a strictly feed-forward explanation based on decision making (Norris et al., 2000).

A clear prediction of the interactive account is that for integration to occur in vOT, it should be functionally connected with other components of the cortical language system during reading. Indeed, previous studies have shown intrinsic functional connections linking vOT with Broca's area (Bitan et al., 2005; Mechelli et al., 2005). Furthermore, recent studies investigating resting-state functional connectivity suggest that a strong intrinsic connectivity exists between Broca's area and ventral occipito-temporal regions even during rest (Koyama et al., 2010; Smith et al., 2009). Thus it was of considerable interest that the activation pattern in POp, a core region of Broca's area, matched that in vOT, suggesting a possible functional linkage between these regions that may contribute to top-down influence on vOT. Confirmation will require evidence of effective connectivity that demonstrates top-down modulation of vOT activity by Broca's area.

Taken together, the current findings demonstrate that activation in vOT during reading is influenced by nonvisual properties of written stimuli and emphasize that interactivity is as important for neural accounts as it is for cognitive and computational models (Coltheart et al., 2001; Harm and Seidenberg, 2004; Jacobs et al., 2003; McClelland and Rumelhart, 1981; Perry et al., 2007; Plaut et al., 1996; Rumelhart and McClelland, 1982). It is worth noting that this conclusion is not specific to reading but rather is in line with a growing literature demonstrating that visual object recognition cannot be a hierarchical, feed-forward process either (Bar et al., 2006; Gazzaley et al., 2007; Gilaie-Dotan et al., 2009; Kveraga et al., 2007; Schrader et al., 2009). These studies challenge the traditional view of serial, bottom-up visual object recognition and instead support non-hierarchical mechanisms which integrate top-down feedback to influence recognition process (see also Bar, 2003; Bullier, 2001; Bullier and Nowak, 1995). Together these studies highlight a need to focus not only on the nature of neuronal representations, but also on the dynamics of this information processing. Critically, this involves elucidating both the functional and anatomical connectivity, which will hopefully help to close the gap between cognitive and neuro-anatomical models of reading.

## Figures and Tables

**Table 1:** Common activations across the four conditions relative to fixation. For each peak in the mean activation contrast, its anatomical location, *Z*-score and standard space (i.e. MNI152) coordinate are displayed. In addition, the *Z*-score at that peak is shown for each of the four individual conditions relative to fixation to illustrate that activation was present for all four conditions.

Region		*Z*-score	Mean peak coordinate			*Z*-score relative to rest			
x	y	z	Orthographic		Phonological	
W	PH_1_	PH_2_	PW
*Occipital*									
L	vOT	11.6	− 44	− 56	− 15	4.5	4.6	5.1	5.1
R	vOT	8.7	45	− 63	− 13	3.5	3.4	3.3	3.9
L	Calcarine sulcus	9.5	− 7	− 76	8	4.0	4.4	4.5	4.0
R	Calcarine sulcus	9.2	9	− 74	12	4.2	4.3	4.3	3.8

*Parietal*									
L	Intra-parietal sulcus	10.2	− 27	− 52	46	3.8	4.1	5.0	4.7
R	Intra-parietal sulcus	9.0	27	− 56	47	4.0	4.4	4.8	4.0
L	Supramarginal gyrus	10.4	− 48	− 33	46	3.6	4.0	4.4	3.7
L	Parietal operculum	8.7	− 54	− 17	18	5.0	3.7	3.4	4.2
L	Postcentral gyrus	10.3	− 40	− 21	50	3.7	4.0	3.1	3.1

*Frontal*									
L	Frontal operculum	8.5	− 31	24	2	3.7	4.3	4.0	4.2
R	Frontal operculum	9.5	33	25	− 3	4.9	4.6	4.5	4.4
L	IFS/PCS junction	11.1	− 42	7	26	4.1	4.4	5.2	4.5
R	IFS/PCS junction	9.3	44	5	28	4.2	3.6	3.3	3.6
L	Pre-SMA	10.6	− 3	15	45	4.8	5.1	5.6	5.3
L	Precentral gyrus	9.1	− 44	− 1	40	3.6	4.5	4.1	4.8

*Subcortical*									
L	Cerebellum (lobe VI)	8.1	− 6	− 73	− 20	3.8	4.5	4.0	4.5
R	Cerebellum (lobe VI)	10.3	21	− 52	− 22	4.7	4.5	4.6	4.3
R	Cerebellum (lobe VI)	10.1	35	− 49	− 23	5.3	4.9	4.3	4.7
R	Cerebellum (lobe VI)	8.8	11	− 25	− 22	4.3	3.9	3.6	4.1
L	Putamen	7.3	− 26	− 1	0	3.6	3.9	3.8	3.9
L	Thalamus (MD)	8.1	− 12	− 18	5	3.8	3.9	3.7	4.8

Abbrev: W = words (orthographic task), PH_1_ = pseudohomophones (orthographic task), PH_2_ = pseudohomophones (phonological task) and PW = pseudowords (phonological task); vOT = ventral occipito-temporal cortex, IFS = inferior frontal sulcus, PCS = precentral sulcus, SMA = supplementary motor area, and MD = mediodorsal nucleus.
